# Psychometric and electrodermal activity data from an experimental paradigm of memory encoding with some items periodically followed by painful electric shock

**DOI:** 10.1016/j.dib.2020.105669

**Published:** 2020-05-08

**Authors:** Ally T. Citro, Caroline M. Norton, Samantha J. Pcola, Keith M. Vogt

**Affiliations:** a Department of Anesthesiology and Perioperative Medicine, University of Pittsburgh, School of Medicine, Pittsburgh, PA, USA; b Department of Bioengineering, Swanson School of Engineering, University of Pittsburgh, Pittsburgh, PA, USA; c Center for the Neural Basis of Cognition, Pittsburgh, PA, USA

**Keywords:** Conditioning, pain, Galvanic skin response, Physiologic response

## Abstract

How pain influences explicit memory is an active area of investigation, and next-day recognition was the primary outcome of this experiment. The data reported here were secondary measures of psychometrics to quantify interindividual variability between subjects and measure electrodermal activity (EDA) changes in response to experimental stimuli. Reliable EDA responses following painful electric shocks were obtained in the Learning portion of the experiment. During next-day testing, however, no reliable EDA responses were elicited, including to previously pain-paired experimental items.

Specifications Table

**Table:** 

Subject	Behavioral Neuroscience
Specific subject area	Pain memory
Type of data	Table, graphs
How data were acquired	Physiologic data was collected using BIOPAC MP160 data acquisition unit and recorded on a Windows 10 PC running AcqKnowledge version 5.0. Psychometric data was collected through written self-report questionnaires including the State Inventory of Cognitive and Somatic Anxiety, Pain Vigilance Assessment Questionnaire, Pain-Anxiety Symptom Scale, and Pain Catastrophizing Scale.
Data format	Raw, summarized
Parameters for data collection	Subjects completed the written questionnaires priors to experiencing any experimental pain. Physiologic data were collected as experimental stimuli were presented in both “Pain” and “No Pain” conditions, as well as during the memory testing experimental session.
Description of data collection	Using a randomized design, words were delivered auditorily in “Pain” and “No Pain” conditions as subjects made decisions about these words. A painful electric shock was administered directly after one third of the words in the Pain condition. Word recognition was tested after 24 hours. Electrodermal activity was measured during all experimental procedures and changes were compared between the words associated with pain vs those that were not.
Data source location	Institution: University of Pittsburgh City/Town/Region: Pittsburgh, Pennsylvania Country: USA
Data accessibility	The raw physiologic data and raw psychometric data is uploaded as a zip file with this article. The analyzed physiologic data is provided within this article.

## Value of the data

• The presented data capture the variation in pain sensitivity and anxiety in healthy volunteer subjects using psychometric questionnaires. This is coupled with physiologic data, including electrodermal activity and electrocardiogram, following experimentally-delivered painful shocks. The data are useful for studying the association between pain and autonomic responses.
• Researchers in psychology and neuroscience, especially those relating pain to conditioned responses and memory of painful experiences, may benefit from this data.
• This information will be useful for designing future experiments concerning auditory stimuli and conditioned pain. It provides insight regarding physiologic measurements of conditioned responses and whether these persist during next-day testing.

## 1 Data Description

Raw data is presented for a series of psychometric questionnaires related to pain. In Table 1, the raw scores for all measures are provided for each subject. Measures include the State Inventory for Cognitive and Somatic Anxiety (CSA), the Pain Anxiety Symptom Scale – Short Form 20 (PASS), the Pain Vigilance and Awareness Questionnaire (PVAQ), and the Pain Catastrophizing Scale (PCS).

**Table 1:** Raw scores for psychometric inventories including the State-Trait Inventory for Cognitive and Somatic Anxiety (CSA), the Pain Anxiety Symptom Scale – Short Form 20 (PASS), the Pain Vigilance and Awareness Questionnaire (PVAQ), and the Pain Catastrophizing Scale (PCS).

Subject #	CSA	PASS	PVAQ	PCS
1	29	49	27	18
2	30	32	43	26
4	27	13	18	7
5	21	20	14	2
6	23	18	35	9
7	21	32	25	18
8	51	47	35	13
9	37	52	27	23
10	29	37	49	9
11	35	45	32	26
12	38	28	17	8
13	22	5	28	7
14	24	17	16	4
15	22	0	6	2
16	23	15	6	0
17	23	30	32	14
18	24	29	28	3
19	31	27	38	15
20	22	13	35	6
21	24	33	27	14
22	28	25	34	12
23	24	4	10	1
24	44	25	35	10
25	21	7	13	2
26	34	35	54	13

Electrodermal activity (EDA) and electrocardiograph data are presented as physiologic responses to experimental stimuli. The raw physiologic data collected during the Learning and Testing sessions for each subject are provided as individual comma-separated value files. Rows in the files represent consecutive time points, with a sampling rate of 625 Hz. Consecutive columns (indicated by alphabetic characters) within these files represent the digitized values for signals, described as follows. Column A contains raw EDA amplitude values over time. Column B is a marker channel that captures a 0 versus 5 Volt square waveform that indicates the timing of shock delivery for pain-paired words in the Learning data files. In the Testing session data files, Column B simply indicates previously pain-paired word timing, as no shocks were delivered. Column C captures a square wave marking the appearance of non-pain words heard in both the Learning and Testing sessions. The upslope of the wave coincides with the termination of the word playing in the Learning files, but, notably, no shock is delivered following these words. Column D is a marker channel for the timing of presentation of new words (foils) in the memory testing files (this channel was not used in the Learning session). Column E contains the raw electrocardiograph tracing, captured from lead II (right arm to left leg) electrodes.

**Figure 1:**
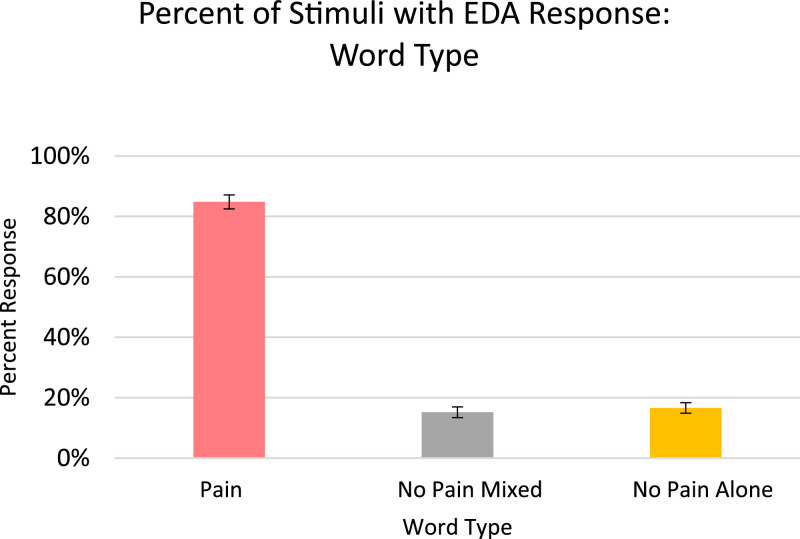
The percent of stimuli followed by an EDA response across all trials and conditions, specified by word type: Pain, No Pain Mixed, or No Pain Alone.

**Figure 2:**
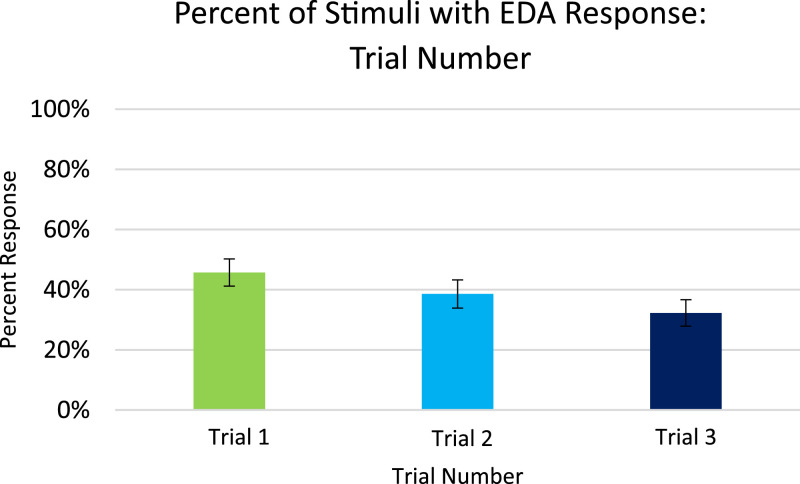
The percent of stimuli followed by an EDA response across all word types and conditions, specified by each of the three trials.

**Figure 3:**
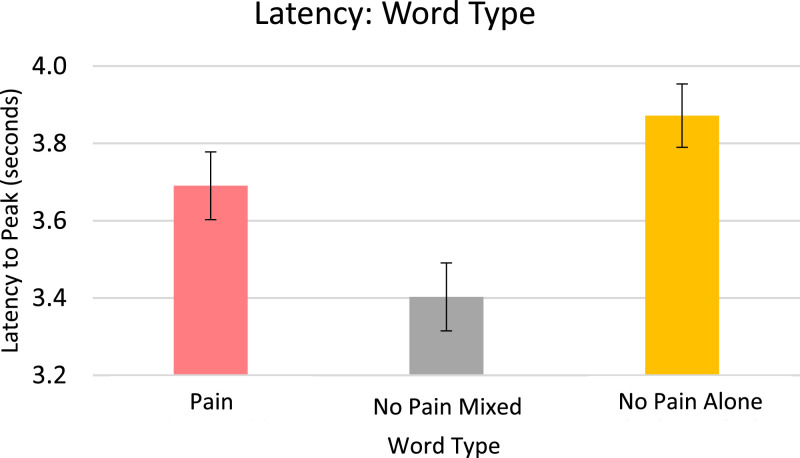
The latency to peak, in seconds, of EDA responses across all trials and conditions, specified by word type: Pain, No Pain Mixed, or No Pain Alone.

**Figure 4:**
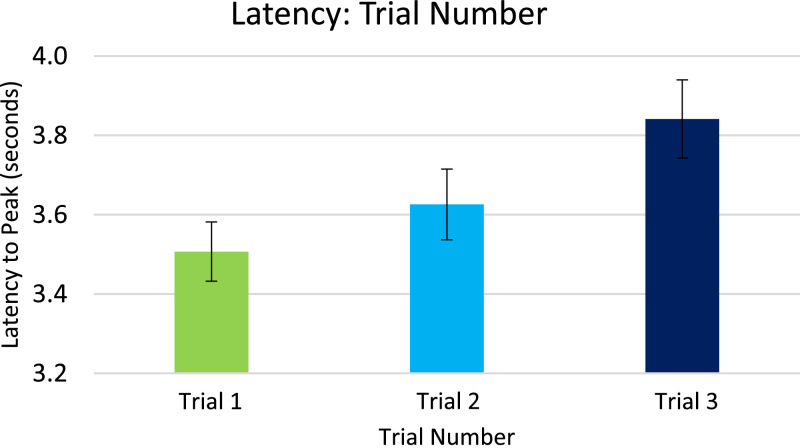
The latency to peak, in seconds, of EDA responses across all word types and conditions, specified by each of the three trials.

**Figure 5:**
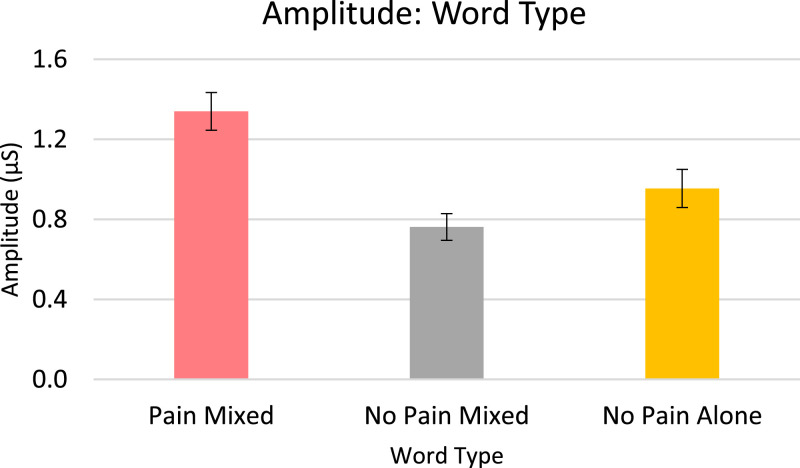
The amplitude, in microSiemens (μS), of EDA responses across all trials and conditions, specified by word type: Pain, No Pain Mixed, or No Pain Alone.

**Figure 6:**
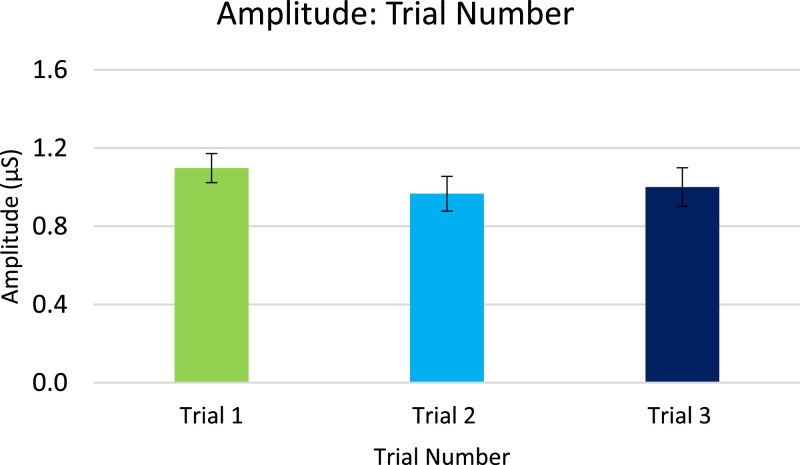
The amplitude, in microSiemens (μS), of EDA responses across all word types and conditions, specified by each of the three trials.

**Figure 7:**
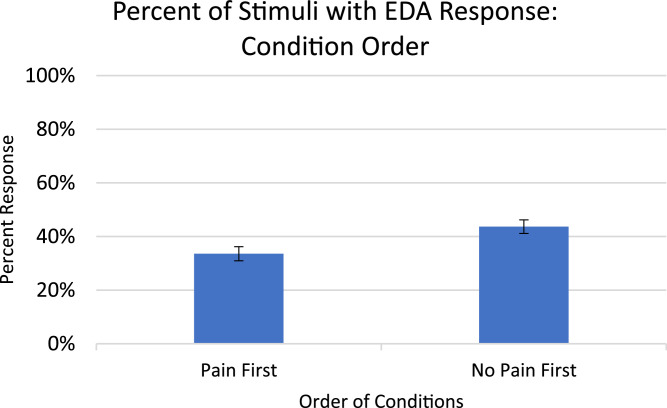
The percent of stimuli followed by an EDA response across all word types, specified by the order of conditions, Pain First or No Pain First.

**Figure 8:**
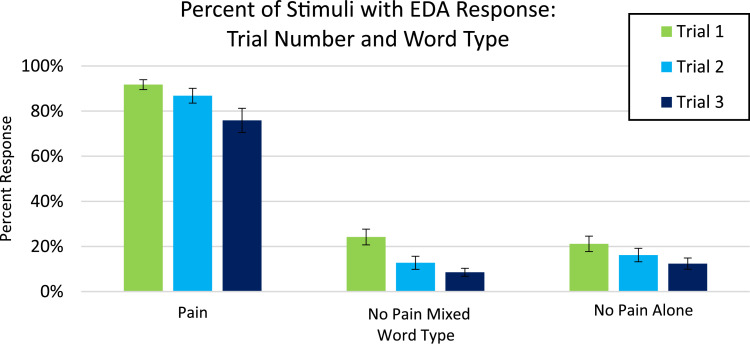
The percent of stimuli followed by an EDA response, specified by each of the three trials and word types.

Analysis for EDA, included in the article, consists of the percent of responses, latency, and amplitude across types of stimuli with various associations to pain. EDA also serves as a measure of learned physiologic response to previously-experienced pain, although this measure did not show differences across stimuli types so analyzed data is only from the Learning session. The percent of EDA responses for all stimuli are depicted according to word type (Fig. 1) and trial number (Fig. 2). The latency, in seconds, to the peak of the responses are shown in Fig. 3 according to word type, and in Fig. 4 according to trial number. The amplitudes are also displayed according to word type (Fig. 5) and trial number (Fig. 6) and measured in microSiemens. Fig. 7 depicts the percent of EDA responses for all stimuli according to condition, either Pain First or No Pain First. The percent of responses, separated by both word type and trial number, are shown in Fig. 8.

## 2 Experimental Design, Materials, and Methods

### 2.1 Design

Subjects completed two hour-long visits, which were respectively called the “Learning” and “Testing” portions. During the Learning session, subjects first completed several psychometric questionnaires to assess traits related to pain such as anxiety, awareness, and sensitivity. For the experiment, subjects heard and responded to auditory stimuli, some of which were associated with pain. Subjects were randomized to receive either the “Pain” or “No Pain” condition first. Electrodermal activity (EDA) was measured while the stimuli were delivered to capture physical responses to painful electric shock. For the Testing portion the following day, subjects heard stimuli from the Learning session mixed with new stimuli. No pain was administered. Subjects’ recognition of stimuli was assessed, while EDA data were again acquired to measure their remembered physiologic response to painful stimuli.

### 2.2 Procedure

#### 2.2.1 Learning Session

During the Learning session, subjects first completed a written informed consent and a battery of psychometric questionnaires. EDA and nerve stimulator electrodes were placed, and the subject was told the nerve stimulator would be titrated to their pain rating for 7/10 on the numeric rating scale (NRS) from 0-10 (0 no pain; 10 worst pain). To accomplish this setting, the investigator turned on the nerve stimulator and collected pain ratings as the current was increased gradually in 1 mA increments. Once a 7/10 on the NRS was reached, subjects received a few shocks of several seconds that mimicked the pain stimuli in the experiment to ensure the current provided the desired pain rating.

The order of the two encoding conditions, Pain and No Pain, was randomized and the experimental design was explained to the subjects. Individual, common words served as stimuli (presented aurally through headphones) and were each .75 seconds in duration with a gap of 2-5 seconds between consecutive words. During the Pain condition, one third of these stimuli were paired with a painful electric shock that lasted 1 second and had an onset immediately after the end of the word. The distribution of pain-paired words and non-pain words in this condition were distributed pseudo-randomly, with constraints that no more than 2 pain-paired words could be consecutive and no more than 5 non-pain words could be consecutive. During the No Pain condition, shocks were not administered.

Both conditions consisted of a separate list of 90 words, in random order, that were repeated in three experimental blocks. Each of the six blocks were presented with a judgement the subject had to make while hearing the list of words, such as Is it living? Does it move? Is it natural? Subjects indicated their response, yes or no, by pressing a number on the keypad. The condition and order the blocks were presented in were both randomized. Before the experimental task began, several practice words were played to ensure the subject's comprehension and an appropriate setting of the nerve stimulator.

#### 2.2.2 Testing Session

The randomized list of 360 total items included all the words played in the Learning portion and an equal number of new words, and subjects were primarily performing an explicit recognition memory task using the Remember-Know-New (RKN) scheme [1]. Full experimental procedures for the memory testing session, and the explicit memory testing data are described in another manuscript [2]. As in the Learning session, the duration of each word was .75 seconds with a several second gap between consecutive words. The following word would not yet be played without an RKN response for the current word. EDA and EKG monitoring were performed during this portion of the experiment to detect any remembered physical responses from words previously associated with a painful stimulus. Although no experimental shocks were delivered, the nerve stimulator and its electrodes were set up in the same manner as in the Learning portion for the subject to feel the same potential threat of experiencing pain.

### 2.3 Materials

#### 2.3.1 Psychometric Questionnaires

To measure the subjective experience of pain, subjects completed a battery of written psychometric questionnaires prior to any pain administration or other data collection. The standardized questionnaires include the State Inventory for Cognitive and Somatic Anxiety (CSA) [3], the Pain Anxiety Symptom Scale – Short Form 20 (PASS) [4], the Pain Vigilance and Awareness Questionnaire (PVAQ) [5], and the Pain Catastrophizing Scale (PCS) [6]. The 21-item CSA captures current physical and emotional characteristics that are typically associated with painful experiences. Responses indicate the degree, ranging from 1 to 4 (1 not at all; 4 very much so) in which a subject agrees with statements such as “My heart beats fast,” or “I think that the worst will happen.” The PASS measures the frequency from 0 to 5 (0 never; 5 always) in which thoughts are present such as “I find it hard to concentrate when I hurt.” The PVAQ includes 16 items, 2 of which are reverse scored, and considers the frequency of behavior over the past 2 weeks. Subjects respond from 0 to 5 (0 never; 5 always) to statements such as “I keep track of my pain level,” and “I am quick to notice changes in location or extent of pain.” Finally, the PCS measures a subject's agreement with 13 statements all beginning with “When I am in pain . . .” and concluding with items such as “I feel I can't go on,” or “I keep thinking about how badly it hurts.” Response options range from 0 to 4 (0 not at all; 4 all the time). Scores for each questionnaire were calculated from summing the responses to individual items. The ranges for possible scores are 21-84 for the CSA, 0-100 for the PASS, 0-80 for the PVAQ, and 0-42 for the PCS. For all inventories, higher scores indicate higher levels of the construct being measured.

#### 2.3.2 Equipment

The experimental procedure was implemented with E-Prime version 2.0 (Psychology Software Tools, Sharpsburg, PA) on a laptop running Windows 10. Subjects heard the experimental items through headphones and indicated their responses on the number keypad. The pairing of words with electric shocks was accomplished using the same custom hardware as our previous investigation [7] that allowed E-Prime to control relays via a serial-emulated USB connection. The nerve stimulator was modified in the same manner, so that the push-button switches on the front of the device could be closed electronically using computer control. A 5 V square-wave logic signal indicating the timing and type of each word being played was generated by a separate bank of relays, also controlled by E-Prime. These trigger signal waveforms were recorded using the BIOPAC MP160 (BIOPAC Systems, Goleta, CA) data acquisition unit and transferred to AcqKnowledge version 5.0 (BIOPAC Systems, Goleta, CA) running on a separate Windows 10 laptop PC. Simultaneous collection allowed for alignment of the experimental events with physiologic data recordings.

#### 2.3.3 Electrodermal Activity

EDA was acquired from the subject's left palm, with EL507 electrodes placed on the hypothenar and thenar eminences. The BIOPAC collected EDA data and transferred it to AcqKnowledge. Pain stimuli were generated using an electric nerve stimulator, with the placement of two electrodes on the subject's left index finger.

### 2.4 Subjects

Healthy volunteer subjects between the ages of 18-30 were recruited from the University of Pittsburgh community using the online Pitt+Me CTSI registry. A total of 26 subjects (16 female) were enrolled, with an average age of 22.0 ± 3.3 years. Eligibility was determined by self-report of exclusionary criteria, with all subjects denying significant memory impairment, hearing loss, sleep apnea, chronic pain, neurologic and psychiatric disease, as well as the use of antidepressants, antipsychotics, antihistamines, anxiolytics, stimulants, sleep aids, and pain medications. Compensation was provided at the rate of $10 per hour. The protocol was approved by the University of Pittsburgh Institutional Review Board (PRO16110197) and conforms to all relevant standards for the ethical and responsible conduct of research.

Of the 26 subjects enrolled, one was lost to follow up and had all data omitted. The average age of the analyzed subjects (15 female) is 21.9 ± 3.3 years. The average nerve stimulatory intensity was 11.4 ± 4.7 mA. After the practice words, subjects reported an average pain rating of 6.38 ± .7 out of 10 on the NRS. Pain ratings following the Pain condition were 6.02 ± 1.0 out of 10. Twelve subjects received the Pain condition first, and 13 received the No Pain condition first.

### 2.5 Electrodermal Activity

EDA data is presented for 21 subjects; four subjects were not included due to excessive movement artifact rendering the data unusable. Of the data presented, 10 subjects were in the Pain First group and 11 were in the No Pain First group. EDA responses were detected for less than 5% of stimuli during the follow-up testing session, therefore the data shown are from the Learning session only.

Electrodermal activity was processed using the same AcqKnowledge software that was used to acquire the data. Peaks in electrodermal responses following the onset of a stimulus (either a pain or non-pain word) were paired to the stimulus if several parameters were met. A 0.05 Hz high pass filter was used with a baseline estimation window width of 1 second. The threshold for responses was 0.03 uS, and responses under 10% of the maximum amplitude were rejected. The minimum separation between the stimulus and event was 0.3 seconds, and the maximum separation was 4 seconds.

For analysis, the presented stimuli were classified into one of three word types: Pain (words presented with a shock in the Pain condition), No Pain Mixed (words not associated with a shock, but presented in the Pain condition), and No Pain Alone (words presented in the No Pain condition). New words (not heard during the Learning portion) were analyzed as a fourth group of stimuli in the Testing data.

## Appendix Supplementary materials

Image, application 1

Image, application 2
